# Cognitive and Emotional Aspects of Cupping Therapy

**DOI:** 10.3390/brainsci10030144

**Published:** 2020-03-04

**Authors:** Minyoung Hong, In-Seon Lee, Yeonhee Ryu, Junsuk Kim, Younbyoung Chae

**Affiliations:** 1 Acupuncture & Meridian Science Research Center, College of Korean Medicine, Kyung Hee University, Seoul 02447, Korea; min.hong27@gmail.com (M.H.); islee4u@gmail.com (I.-S.L.); 2 KM Fundamental Research Division, Korea Institute of Oriental Medicine, Daejeon 34054, Korea; yhryu@kiom.re.kr; 3 Department of Industrial ICT Engineering, Dong-Eui University, Busan 47227, Korea; junsuk.kim5@gmail.com

**Keywords:** arousal, cupping, emotion, motivation, valence

## Abstract

Cupping therapy has recently gained public attention and is widely used in many regions. Some patients are resistant to being treated with cupping therapy, as visually unpleasant marks on the skin may elicit negative reactions. This study aimed to identify the cognitive and emotional components of cupping therapy. Twenty-five healthy volunteers were presented with emotionally evocative visual stimuli representing fear, disgust, happiness, neutral emotion, and cupping, along with control images. Participants evaluated the valence and arousal level of each stimulus. Before the experiment, they completed the Fear of Pain Questionnaire-III. In two-dimensional affective space, emotional arousal increases as hedonic valence ratings become increasingly pleasant or unpleasant. Cupping therapy images were more unpleasant and more arousing than the control images. Cluster analysis showed that the response to cupping therapy images had emotional characteristics similar to those for fear images. Individuals with a greater fear of pain rated cupping therapy images as more unpleasant and more arousing. Psychophysical analysis showed that individuals experienced unpleasant and aroused emotional states in response to the cupping therapy images. Our findings suggest that cupping therapy might be associated with unpleasant-defensive motivation and motivational activation. Determining the emotional components of cupping therapy would help clinicians and researchers to understand the intrinsic effects of cupping therapy.

## 1. Introduction

Cupping therapy, one of the oldest documented medical techniques, is widely used in some regions, including China, India, Korea, the Middle East, and parts of Europe [1,2]. The common denominator in all cupping techniques is the use of suction on the body, achieved by creating a vacuum [3]. The negative pressure induced by cupping therapy causes stretching of the skin and underlying tissue, and dilation of the capillaries [4,5]. This process induces enhanced microcirculation, tissue detoxification, and relief from painful muscle tension [6,7]. A systematic review suggested that cupping therapy might be beneficial for a variety of conditions, particularly herpes zoster, acne, facial paralysis, and cervical spondylosis [8]. However, most clinical trials have been of poor quality and have yielded no firm conclusions [6]. 

Recently, cupping therapy has gained in popularity and attracted the attention of the public, with extensive media coverage of the circular bruises left on Michael Phelps’ shoulders and back at the 2016 Rio de Janeiro Olympics [9]. One concern regarding cupping therapy has been the potential transmission of blood-borne infections, including hepatitis C [10]. Severe or life-threatening side effects from cupping therapy have been reported, although such adverse events are extremely rare when cupping techniques are performed with care [11,12,13]. Cupping therapy may produce a circular patch characterised by redness, petechiae, and ecchymoses, or bruising, but these are not typically severe and generally disappear after 1–2 weeks [14,15,16]. Nevertheless, some patients are still afraid of being treated with cupping therapy and express concerns about the visually unpleasant marks that it leaves. 

All medical treatments inevitably include both physiological and psychological components, and it is well known that patients’ symptoms can be influenced by the contextual or environmental cues associated with medical interventions [17]. Patients’ beliefs and expectations can markedly influence the therapeutic benefits and adverse effects of a treatment [18]. Positive expectations of the treatment can enhance its therapeutic efficacy, while negative expectations can diminish it [19]. Prior expectations about acupuncture treatment were associated with significant improvement in patients with chronic pain [20]. As with other medical treatments, positive and/or negative expectations can influence the effectiveness of cupping therapy. 

A cognitive–emotional model of the placebo effect proposes that positive suggestions about improvements in physical health lead individuals to attend selectively to signs of improvement [21]. The motivational model describes emotional states as having two major components: hedonic valence (pleasant appetitive motivation or unpleasant defensive motivation), and arousal (degree of motivational activation) [22]. Placebo responses are related to positive feelings about the prospects of relief or pleasure associated with treatments [23]. The nocebo effect, by contrast, is caused by negative expectations and the associated fearful and stressful emotions [24]. To understand the intrinsic action of cupping therapy, therefore, it is necessary to determine its valence, and the arousal level for patients. 

Hence, the purpose of the current study was to examine the cognitive and emotional components of cupping therapy. Participants provided valence and arousal ratings immediately after the presentation of visual stimuli illustrating fear, disgust, happiness, neutral emotion, and cupping therapy, along with control images. We assessed the spatial organisation of participants’ responses in two-dimensional psychophysical space, based on their responses to the emotional stimuli.

## 2. Materials and Methods

### 2.1. Participants

In total, 25 healthy volunteers were recruited via online advertisements directed at students attending Kyung Hee University and Korea University in Seoul, Republic of Korea. None of the participants had any history of neurological, cardiac, psychiatric, or other major medical problems. Participants were prohibited from using medication, alcohol, or caffeine for 12 h prior to the experiment. All participants received a detailed explanation of the experimental procedure and provided written informed consent. This experiment was conducted in accordance with the Declaration of Helsinki and approved by the Institutional Review Board of Kyung Hee University (KHSIRB-18-075).

### 2.2. Emotional Visual Stimuli 

The experimental stimuli were selected from the International Affective Picture System (IAPS) [22]. Four different kinds of affective images were derived from the IAPS: fear, disgust, happiness, and neutral emotion (IAPS catalogue numbers: fear (*n* = 10): 1301, 1303, 1726, 5940, 6211, 6244, 6370, 6825, 7640, 9620; disgust (*n* = 10): 1111, 7359, 7361, 7380, 9140, 9300, 9301, 9500, 9570, 9571; happiness (*n* = 10): 1340, 2216, 2345, 2391, 5600, 5833, 8170, 8190, 8496, 8540; neutral emotion (*n* = 10): 2383, 5535, 7037, 7130, 7186, 7211, 7495, 7503, 7510, 7595). The cupping therapy images (*n* = 16) and control images (*n* = 4) were made in-house. The cupping therapy images included several circular patches with redness, petechiae and ecchymoses, or bruising on body areas. Each of the cupping therapy images were paired with a control image that was matched as closely as possible for visual characteristics (e.g., colour, brightness, background scene). The control images were shown with the same body areas, except cupping therapy images. All pictures were approximately 20.7 inches wide and 11.5 inches high when displayed centrally on a 27-inch monitor. The order of presentation was randomised across the participants. The experiments were conducted in an air-conditioned (24 ± 2 °C), soundproofed room.

### 2.3. Experimental Procedure

Participants were seated in a comfortable armchair at a constant distance of about 70 cm from the monitor. They were instructed to look at the fixation cross at the start of each trial. To minimise any potential noise from the environment, earmuffs were worn throughout the experiment. Participants were told that they would be presented with 60 images containing emotional components. 

**Figure 1:**
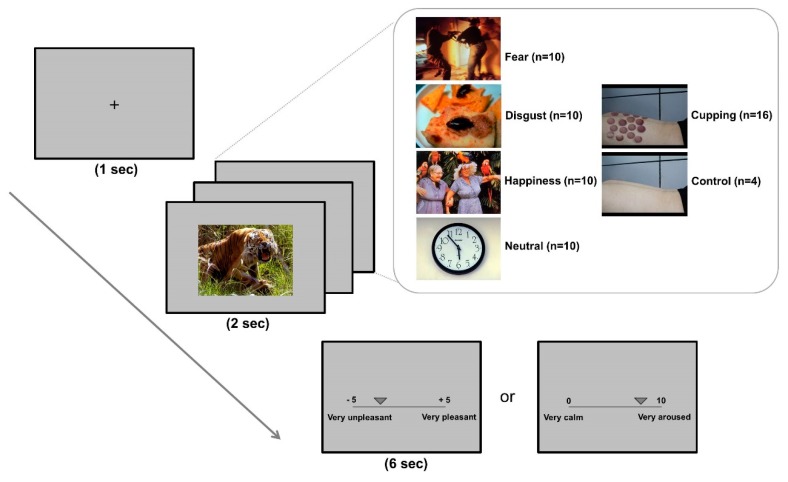
Experimental design. Four different kinds of affective images (fear, disgust, happiness, neutral) were taken from the International Affective Picture System (IAPS). The cupping therapy images included images of several circular body areas with patches of redness, petechiae and ecchymoses, or bruising. Control images of body areas were matched as closely as possible for visual characteristics (e.g., colour, brightness, background scene). At the start of each trial, a fixation cross was displayed in the centre of the screen for 1000 ms. The fixation cross was followed by emotional stimuli, including IAPS and cupping therapy images, which were displayed for 2000 ms. After the presentation, participants were given 6000 ms to evaluate the valence and arousal level of each emotional stimulus. Using a computerised visual analogue scale, participants moved a button along a bar to indicate the valence or arousal value (valence: −5 = *very unpleasant*, 5= *very pleasant*; arousal: 0 = *very calm*, 100 = *very aroused*).

At the start of each trial, the fixation cross was displayed in the centre of the screen for 1000 ms. The fixation cross was followed by emotional stimuli, including IAPS and cupping therapy images, for 2000 ms. After each stimulus presentation, participants were given 6000 ms to evaluate the valence and arousal level of the emotional stimulus. A 2000-ms rest period was provided between trials. To rate the stimuli, participants used a computerised visual analogue scale, moving a button along a bar on the monitor to indicate the appropriate valence and arousal values (valence: −5 = *very unpleasant*, 5 = *very pleasant*; arousal: 0 = *very calm*, 10 = *very aroused*). To minimise the cognitive load, the valence and arousal ratings were completed separately in two sessions (Figure 1). 

### 2.4. Fear of Pain Questionnaire

All participants were asked to complete the Fear of Pain Questionnaire-III (FPQ-III) before the experiment. FPQ-III, which addresses fear of “severe”, “minor”, and “medical” pain, was developed to assess an individual’s fear of a variety of different stimuli that may induce pain [25].

### 2.5. Data Analysis

Values are expressed as means ± standard error (SDE). The arousal and valence ratings were averaged within each visual stimulus. Behavioural data were analysed using one-way analysis of variance (ANOVA). Differences between the cupping therapy images and the control images were analysed using a paired *t*-test. Statistical analyses were performed using R software (ver. 3.4.4.; R Development Core Team, Vienna, Austria). Pearson’s correlation analysis was also performed to determine the correlation between the arousal level of cupping therapy images and FPQ-III scores. The level of significance was set at *p* < 0.05 for all analyses.

The calculated mean valence and arousal ratings of the 60 images were mapped on the *x*- and *y*-axes to establish the two-dimensional psychophysical space. To assess grouping patterns, we employed *K*-means clustering algorithms. *K*-means clustering is an unsupervised machine learning algorithm that partitions a dataset into a user-specified number (*K*) of clusters. Clusters were formed based on the sum of point-to-cluster-centroid Euclidean distances. Moreover, to determine the optimal number of clusters, the error was calculated as the within-cluster sum of point-to-centroid distances summed over different values of *K*. We searched for the *K* value with which there was no substantial change in the rate of error decrease (i.e., the elbow method) [26,27]. 

## 3. Results

### 3.1. Baseline Characteristics

In total, 25 participants (14 females; age = 24.6 ± 0.8 years) took part in this study. Four participants had prior experience with cupping therapy. 

### 3.2. Psychophysical Responses to Emotional Visual Stimuli

**Figure 2:**
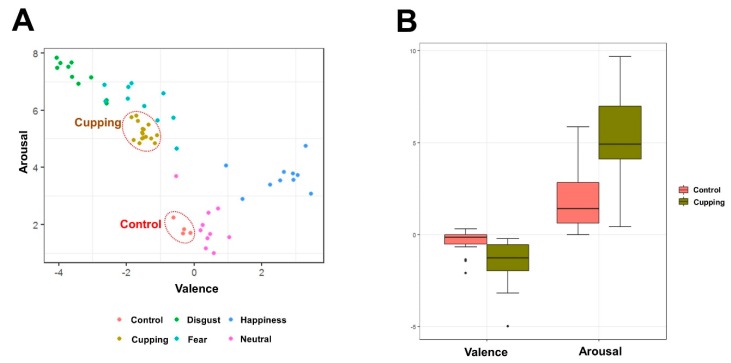
Valence and arousal ratings of visual stimuli. **A**: Each image was plotted in two-dimensional affective space, as defined by its mean valence and arousal ratings. Emotional arousal increases as hedonic valence ratings become increasingly more pleasant or unpleasant. **B**: Comparison of the valence and arousal ratings in response to the cupping therapy images and the control images. Cupping therapy images were rated as more unpleasant and more arousing than control images.

Valence ratings (F _(6,144)_ = 158.832, *p* < 0.001) and arousal ratings (F _(6,144)_ = 36.163, *p* < 0.001) differed significantly among the stimulated emotional visual images. Each image was plotted in the two-dimensional space defined by its mean valence and arousal ratings. When valence ratings were plotted on the *x*-axis and arousal ratings on the *y*-axis, the quadratic relationship was stronger between the valence and arousal ratings. Emotional arousal increased as hedonic valence ratings became increasingly more pleasant or unpleasant (Figure 2A). 

We compared the valence and arousal ratings between the cupping therapy and control images (Figure 2B). Cupping therapy images showed significantly lower valence ratings (−1.50 ± 0.24 *vs.* −0.33 ± 0.11, *t* = 6.118, *p* < 0.001) and significantly higher arousal ratings (5.22 ± 0.48 vs. 1.87 ± 0.32, *t* = 7.877, *p* < 0.001) compared with the control images.

### 3.3. Cluster Analysis of Emotional Visual Stimuli

**Figure 3:**
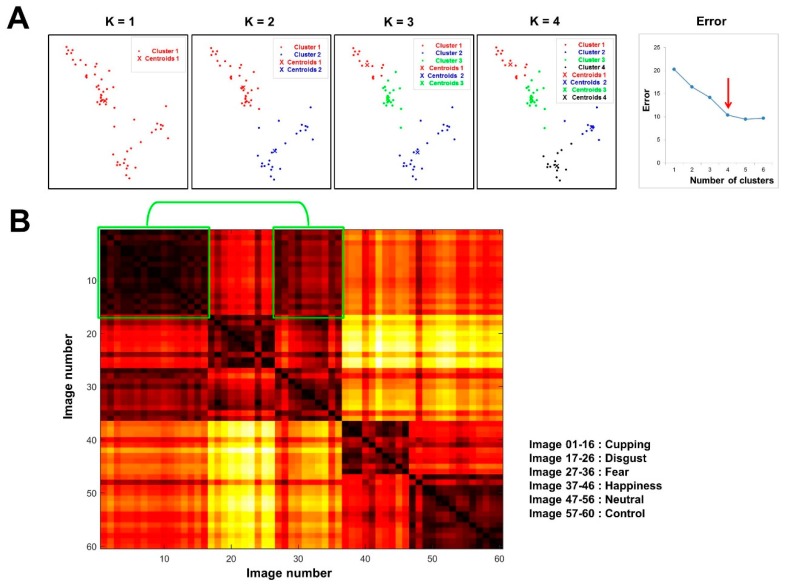
Cluster analysis of visual stimuli. **A**: Cluster analysis using the *K*-means clustering method. As *K* changed from one to six, the errors were 20.23, 16.45, 14.16, 10.37, 9.43, and 9.66, respectively. Setting the number of clusters at four yielded a compact spatial organisation. Most of the cupping therapy images were grouped together with fear images, whereas the control images were grouped together with neutral images. **B**: The two-dimensional spatial organisation of psychophysical responses to the emotional stimuli. Cupping therapy images were separated from fear images by shorter distances (in the green box), but from happy, neutral, and control images by longer distances. The image pairs separated by small distances are coloured a darker red than those separated by larger distances.

Once we obtained a spatial depiction of the 60 images, we applied *K*-means clustering (Figure 3A). First, to determine the optimal number of clusters (i.e., *K*), we estimated how close the points were to one another in each cluster across different values of *K*. As *K* changed from one to six, the errors were 20.23, 16.45, 14.16, 10.37, 9.43, and 9.66, respectively. According to the location of a bend in the plot, we concluded that setting the number of clusters to four yielded a compact spatial organisation. In this organisation, most of the cupping therapy images were grouped together with fear images, whereas control images were grouped together with neutral images.

Moreover, we assessed the spatial organisation of the two-dimensional psychophysical responses to emotional stimuli (Figure 3B). The distances separating all possible image pairs were calculated based on the sum of the Euclidean distances, and a distance matrix was constructed accordingly. The distance separating each pair indicated the similarity of psychophysical patterns between the images. The results of the image clustering were further supported by this distance map of emotional images. Overall, images belonging to the same category tended to be separated by small distances, whereas those in different categories were separated by larger distances. Notably, cupping therapy images were relatively close to fear images, but farther from happiness, neutral, and control images. 

### 3.4. Correlation Between Emotional Components of Cupping Therapy and Fear of Pain Questionnaire Scores

**Figure 4:**
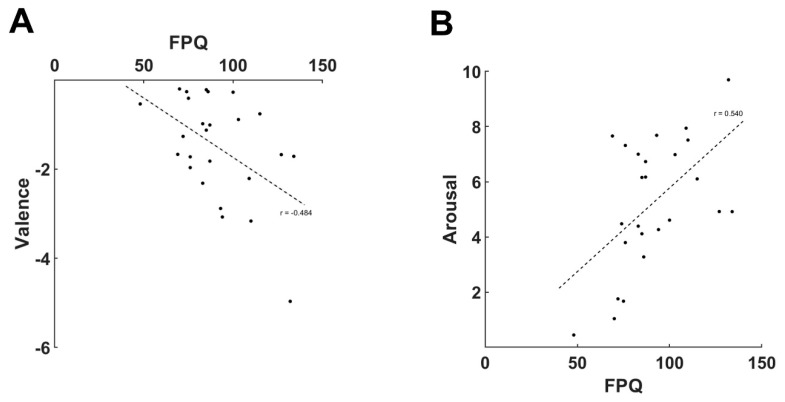
Correlation analysis between the emotional components of cupping therapy and scores on the Fear of Pain Questionnaire. Greater fear of pain was associated with increased feelings of unpleasantness and arousal in response to cupping therapy images, **A**: Correlation analysis between the valence ratings for cupping therapy and FPQ scores. **B**: Correlation analysis between the arousal ratings for cupping therapy and FPQ scores.

We found a significant negative correlation between the valence ratings for cupping therapy and FPQ scores (*r* = −0.484, *p* < 0.05) (Figure 4A), and a significant positive correlation between the arousal ratings for cupping therapy and FPQ scores (*r* = 0.540, *p* < 0.01) (Figure 4B). 

## 4. Discussion

Our aim in this study was to identify the cognitive and emotional components of cupping therapy using the IAPS system. We demonstrated that cupping therapy images were regarded as more unpleasant and arousal-inducing than the control images. Cluster analysis showed that cupping therapy elicited emotional responses similar to those for fear images. Individuals with a greater fear of pain rated cupping therapy images as more unpleasant and more arousing. These findings suggest that cupping therapy might be closely associated with unpleasant–defensive motivation and motivational activation. 

The hedonic valence (appetite or defence) and arousal dimensions have been considered the strategic dimensions of the emotional world [28]. The IAPS, a large set of emotionally evocative colour photographs, can provide experimental control stimuli and emotional stimuli for cognitive neuroscience research [29]. Brief exposure to these standardised images led participants to report emotional experiences that varied reliably along dimensions of pleasure and emotional arousal [22]. In this study, both valence and arousal ratings were significantly different across the visual images in the two-dimensional affective space. Similar to the U-shaped distributions reported in previous studies, emotional arousal in the present study increased as hedonic valence ratings became increasingly more pleasant or unpleasant. Unpleasant stimuli, such as those that evoke fear and disgust, range from calming to highly arousing on the left-hand side of the psychophysical space (defensive motivation), while pleasant stimuli, such as those that evoke happiness, similarly range from calming to highly arousing on the right-hand side (appetitive motivation). These findings are consistent with previous studies in which judgments of pleasure or displeasure indicated that the motivational system was active, and judgments of arousal indicated the intensity of that motivational activation [22]. 

In the current study, cupping therapy images were more unpleasant and arousing than the control images. This suggests that cupping therapy might induce unpleasant–defensive motivation and motivational activation. Because cupping therapy indeed appears to elicit more unpleasant and more aroused emotional states, exposure to this medical intervention might generate more negative expectations about its usefulness. Such expectations not only reduce treatment efficacy, but also influence the occurrence of unwanted adverse effects [30]. Nocebo effects are negative effects on treatment efficacy and tolerability driven by psychological factors or prior experience [31]. Negative information may result from social learning and may even reside outside of conscious awareness [32,33,34]. In our previous study, participants in the group who had negative expectations reported more unpleasant emotions and greater arousal in response to acupuncture pictures than did those in the positive group [35]. Recently, placebo and nocebo effects have been interpreted as predictive coding, i.e., a probabilistic integration of learning and prior knowledge [36]. It is expected that strong adverse emotions toward cupping therapy might be an obstacle to understanding its intrinsic action. Although we did not examine nocebo effects in cupping therapy in the current study, it is important to be aware of these emotional biases and their role in negative-defensive motivation. 

In this study, cluster analysis revealed that most cupping therapy images were grouped together with fear images. The organisation of responses to emotional stimuli in two-dimensional psychophysical space also demonstrated that cupping therapy elicited emotional responses similar to responses to fear images. The Fear of Pain Questionnaire-III, which measures fear of “severe”, “minor”, and “medical” pain, assesses an individuals’ fear of a variety of different stimuli [25]. The Fear of Dental Pain and Fear of Acupuncture questionnaires were developed to examine the roles of fear and pain in clinical settings [37,38]. Our previous study demonstrated that participants who were more fearful of acupuncture-induced pain showed enhanced physiological arousal to the physical stimulation of acupuncture, as measured by skin conductance [39]. Consistent with this finding, the current study showed that participants with a greater fear of pain had more unpleasant feelings and greater arousal in response to images of cupping therapy than those with less fear of pain. For better patient management in clinical settings, it is important to determine whether patients are afraid of pain, and whether they hold an extreme negative view toward a certain medical treatment. Understanding the emotional responses to cupping therapy would be useful for dealing with the aversion to this treatment, and for efforts to minimise unwanted adverse events in a clinical setting. 

This study has some limitations. First, the sample size was small, and the participants had a narrow age range; thus, they might not constitute a representative sample. Studies with larger samples and greater statistical power are necessary in the future. Second, the experienced group showed less unpleasant (−0.84 ± 0.32 *vs.* −1.62 ± 0.26) and less arousal (3.34 ± 1.57 *vs.* 5.58 ± 0.46) emotional responses when we conducted a subgroup analysis between the two groups. However, since we included only four participants who had experienced cupping therapy, it is difficult to confirm that prior experience with cupping therapy contributes to emotional responses in this study. In order to examine the role of experience of cupping therapy, it will be necessary to include more experienced participants, and to compare them with naïve participants in future studies. Finally, since we did not include other medical interventions, we cannot confirm that these negative biases are unique to cupping therapy. Further research is needed to compare cupping therapy with other medical interventions. 

In summary, the current study demonstrated that individuals exhibit elevated negative feelings and increased arousal in response to stimuli depicting cupping therapy. We also suggest that the emotional valence and arousal associated with cupping therapy are, by extension, associated with the fear of pain. Identifying the emotional responses to cupping therapy would help clinicians and researchers to understand the therapy’s intrinsic action. Given the importance of the psychosocial factors associated with medical treatments, the current investigation of the emotional components of cupping therapy may help us to avoid potential unwanted adverse effects and enhance patient adherence and compliance.
